# Genome Sequences of *Erwinia* Phyllophages AH04 and AH06

**DOI:** 10.1128/MRA.00820-21

**Published:** 2021-11-04

**Authors:** Greg P. Krukonis, Sam J. Roth, Véronique A. Delesalle

**Affiliations:** a Department of Biology, Angelo State University, San Angelo, Texas, USA; b Department of Biology, Gettysburg College, Gettysburg, Pennsylvania, USA; DOE Joint Genome Institute

## Abstract

Although crucial in shaping bacterial communities, few bacteriophages of the phyllosphere have been described. We provide genome data for two *Myoviridae* phages, AH04 and AH06, isolated on Erwinia billingiae strains. AH04 shares limited genetic similarity with previously described phages, while AH06 shares over 75% similarity with various *Erwinia* phages.

## ANNOUNCEMENT

Despite their relevance to bacterial population dynamics on plants (1–5), bacteriophages that infect plant pathogens are poorly described. Here, we describe two phages isolated on Erwinia billingiae strains, themselves isolated from the leaves of horse chestnut trees (Aesculus hippocastanum; Sapindaceae) from the same location in Oxford, UK (1–3). The bacterial strains were classified based on sequencing of 800 bp of the 16S rRNA region and the top BLASTn hits associated with a sequence (E value, <10^−10^) (1).

Each phage was single-plaque purified at least three times on its focal host and amplified by overnight culturing in 10 ml King’s broth and 100 μl of isolation bacteria (1). The cultured lysate was filtered (pore size, 0.45 μm), and following the Promega Wizard PCR Preps DNA purification system kit protocol (no. 7170), phage DNA was extracted by the Koskella lab. DNA samples were sent to North Carolina State University’s Genomic Science Laboratory for sequencing. Libraries were prepared using the Illumina TruSeq Nano DNA library prep kit following the manufacturer’s protocol. Sequencing was conducted on the Illumina MiSeq platform, using a v3 150 SE flow cell. For each sample, 150-bp reads were assembled into one contig using the GS v2.9 *de novo* assembler, with >200× coverage (Table 1); the quality of the consensus contig was verified using Consed v29 (6, 7). The genome ends were determined to be circularly permuted through analysis with PAUSE and PhageTerm (8, 9). The sequences were imported into DNA Master v5.22.22 (10) to map the open reading frames. Putative genes were called based on Glimmer v3.0 and GeneMark v2.5 algorithms (11, 12). Putative functions of the gene products were predicted using BLAST v2.12 (13) and HHpred (14). For the BLASTp matches, an E value below 10^−5^ was required to assign a function. For the HHpred matches, a high probability (>85%), substantial coverage (>50%), and low E value (<10^−5^) were required. The presence of tRNA genes was verified through the Web-based program ARAGORN (15). Default settings were used in all analyses.

**TABLE 1:** Isolation information and genome characteristics for *Erwinia* phages AH04 and AH06

Phage name	Isolation yr	No. of reads	Coverage (×)	Genome size (bp)	%GC	No. of protein genes	No. of tRNA genes^*a*^	Best BLASTn matches (GenBank accession no.)^*b*^
AH04	2011	910,482	520	262,639	43.3	293	1; Cys (gca)	Klebsiella phage N1M2 (MN642089.1), Pseudomonas phage OBP (JN627160.1), *Edwardsiella* virus pEt-SU (NC_048182.1)
AH06	2012	400,092	218	275,293	48.1	333	0	vB_EamM_Simmy50 (NC_041974.1), vB_EamM_Special (NC_041975.1), Ea35-70 (KF806589.1)

a The tRNA gene is listed with amino acid (anticodon) information.

b The complete genome of each phage was searched using BLASTn against the nucleotide (nt) database restricted to phages (taxid: 10699, 10662, and 10744). For AH04, matches with more than 40% coverage of the query are reported. For AH06, only the top three matches out of 10 matches with over 80% coverage, all to *Erwinia* phages, are listed.

Both phages have similar GC contents and relatively large genomes, with more than 290 genes, including one tRNA gene for AH04 (Table 1). Based on a BLASTn search of the nucleotide (nt) database restricted to phages (taxid 10699, 10662, and 10744), both phages are likely *Myoviridae*. AH04 shows limited nucleotide similarity (15 to 25%) to three *Myoviridae* phages isolated on different *Proteobacteria* hosts (Table 1). AH06 exhibits greater nucleotide similarity (>75%) to a number of *Myoviridae Erwinia* phages (Table 1). As is typical of *Myoviridae* genomes (16, 17), there is little conservation of genome organization, and only 19 to 20% of genes could be assigned a function. Both genomes include three endolysins, including one with a family 19 chitinase domain—the biggest gene in each genome (7,215 and 6,678 bp, respectively, in AH04 and AH06), which is impressively long, given the average gene length in these phages (851 and 773 bp) and the published average phage gene length of 616 bp (18). Based on sequencing of DNA extracted from different samples, AH04 was isolated twice from different leaves of tree 1 in 2011, while AH06 was isolated three times from the leaves of tree 6 in 2012 (1).

### Data availability.

The genome sequences and associated information can be found under GenBank and SRA accession no. MZ501267 and SRX11736855 (AH04) and MZ501268 and SRX11736857 (AH06), and are also associated with BioProject accession no. PRJNA754193.
